# Supplementary material for: Coral Skeletal Cores as Windows Into Past Symbiodiniaceae Community Dynamics
Source: Glob Chang Biol. 2025 Nov 7;31(11):e70575. doi: 10.1111/gcb.70575 (PMC12592972; doi:10.1111/gcb.70575)
Supplement: Supplementary file 1 — Table S1: Post‐MED sequence number of each core fragment for P. lobata and D. heliopora . Table S2: Pairwise statistical test of differences in the reconstructed Symbiodiniaceae. Table S3: Statistical test of multivariate dispersion in the reconstructed Symbiodiniaceae. Table S4: Statistical test of differences in the reconstructed Symbiodiniaceae communities of. Table S5: Pairwise statistical test of differences in the reconstructed Symbiodiniaceae. Table S6: Statistical test of multivariate dispersion in the reconstructed Symbiodiniaceae. Table S7: Mantel test performed on Symbiodiniaceae community similarities calculated with. Figure S1: Photographs of the coral skeleton cores. (A) Halved coral core of Porites lobata . Figure S2: Distance‐decay linear regression fitted on community similarity Bray‐Curtis. Figure S3: Number of shared post‐MED sequences between P. lobata and D. heliopora . Figure S4: Daily sea surface temperature and days with bleaching alerts for the sampling. [file GCB-31-e70575-s001.pdf]

Supplementary Material

for

Coral skeletal cores as windows into past Symbiodiniaceae community dynamics

Jose F. Grillo[1], Vanessa Tirpitz[1], Jessica Reichert[1,2], Marine Canesi[3], Stéphanie Reynaud[4], Eric Douville[3], Maren Ziegler[1*]

[1] Marine Holobiomics Lab, Department of Animal Ecology & Systematics, Justus Liebig University Giessen, Germany

[2] Hawai‘i Institute of Marine Biology, University of Hawai‘i at Mānoa, Hawai‘i, Kāne‘ohe, USA

[3] Laboratoire des Sciences du Climat et de l'Environnement, CEA-CNRS-UVSQ, Université Paris-Saclay, Gif-sur-Yvette, France

[4] Centre Scientifique de Monaco, Principality of Monaco, Monaco

* Corresponding author: Maren.Ziegler@bio.uni-giessen.de

1. Supplementary tables

Table S1. Post-MED sequence number of each core fragment for *P. lobata* and *D. heliopora* sampled in Palau and Papua New Guinea (PNG). The counts represent the remaining post-MED sequences kept after blank correction.

Sample	Fragment age	Post-MED sequences	Sample	Fragment age	Post-MED sequences
Palau: *P. lobata*	0	59784	PNG: *P. lobata*	0	35064
	5	28479		5	24862
	10	36229		10	36820
	15	29205		15	33140
	20	33067		20	37420
	25	36417		25	54964
	30	33104		30	34415
	80	33654		80	24150
	85	29402		85	35839
	90	38003		90	29501
Palau: *D. heliopora*	0	108490	PNG: *D. heliopora*	0	54359
	5	22595		5	35561
	10	30545		10	42582
	15	22357		15	41390
	20	21556		20	24405
	25	55444		25	42712
	30	110234		30	40984
	100	31621		100	42819
	105	37108		105	16940
	110	44270		110	42416

Table S2. Pairwise statistical test of differences in the reconstructed Symbiodiniaceae communities obtained with three DNA extraction protocols from a *P. lobata* core sampled in Papua New Guinea. Pairwise PERMANOVA on Bray-Curtis distances with 999 permutations. DF: degrees of freedom, SS: sum of squares, F value: F statistic.

Group comparison	Df	SS	F value	R^2	Adj. p-value
PowerSoil vs. Decalcification PowerSoil	1	0.7899	1.8234	0.0920	0.003
PowerSoil vs. Decalcification Ancient DNA	1	0.8508	2.0162	0.1007	0.006
Decalcification PowerSoil vs. Decalcification Ancient DNA	1	0.6222	1.4443	0.0743	0.012

Table S3. Statistical test of multivariate dispersion in the reconstructed Symbiodiniaceae communities obtained with three DNA extraction protocols from a *P. lobata* core sampled in Papua New Guinea. Betadisper on Bray-Curtis distances. DF: degrees of freedom, SS: sum of squares, F value: F statistic.

Factor	Df	SS	Mean Sq	F value	p-value
Groups	2	0.001527	0.00076364	0.5064	0.6083
Residual	27	0.040717	0.00150805		
Total	29	0.042244			

Table S4. Statistical test of differences in the reconstructed Symbiodiniaceae communities of *P. lobata* and *D. heliopora* sampled in Palau and Papua New Guinea. PERMANOVA on Bray-Curtis distances with 999 permutations. DF: degrees of freedom, SS: sum of squares, Pseudo-F: pseudo-F statistic.

**Factor**	**Df**	**SS**	**R^2**	**Pseudo-F**	**p-value**
Coral species	1	0.6966	0.0407	1.6932	0.0001
Sampling location	1	0.6720	0.0393	1.6334	0.0001
Species x Location	1	0.9176	0.0537	2.2304	0.0001
Residual	36	14.8110	0.8663		
Total	39	17.0972	1.0000		

Table S5. Pairwise statistical test of differences in the reconstructed Symbiodiniaceae communities of *P. lobata* and *D. heliopora* sampled in Palau and Papua New Guinea. Pairwise PERMANOVA on Bray-Curtis distances with 999 permutations. DF: degrees of freedom, SS: sum of squares, F value: F statistic.

Group comparison	Df	SS	F value	R^2	Adj. p-value
Palau - *P. lobata* vs. Palau - *D. heliopora*	1	0.9644	2.4318	0.1190	0.006
Palau - *P. lobata* vs. PNG - *P. lobata*	1	0.8796	2.1690	0.1075	0.012
Palau - *D. heliopora* vs. PNG - *D. heliopora*	1	0.7347	1.7604	0.0891	0.006
PNG - *P. lobata* vs. PNG - *D. heliopora*	1	0.6253	1.4669	0.0754	0.006

Table S6. Statistical test of multivariate dispersion in the reconstructed Symbiodiniaceae communities of *P. lobata* and *D. heliopora* sampled in Palau and Papua New Guinea. Betadisper on Bray-Curtis distances. DF: degrees of freedom, SS: sum of squares, F value: F statistic.

Factor	Df	SS	Mean Sq	F value	p-value
Groups	3	0.0173	0.0058	3.9191	0.0161
Residual	36	0.0530	0.0015		
Total	39	0.0703			

Table S7. Mantel test performed on Symbiodiniaceae community similarities calculated with a Bray-Curtis distance matrix and fragment age calculated as a Euclidean distance matrix.

**Location**	**Coral species**	**Mantel Statistic (r)**	**p-value**
Palau	*Porites lobata*	-0.4088	0.9610
Palau	*Diploastrea helipora*	0.3229	0.057
Papua New Guinea	*Porites lobata*	-0.0923	0.633
Papua New Guinea	*Diploastrea helipora*	-0.1598	0.7220

2. Supplementary figures

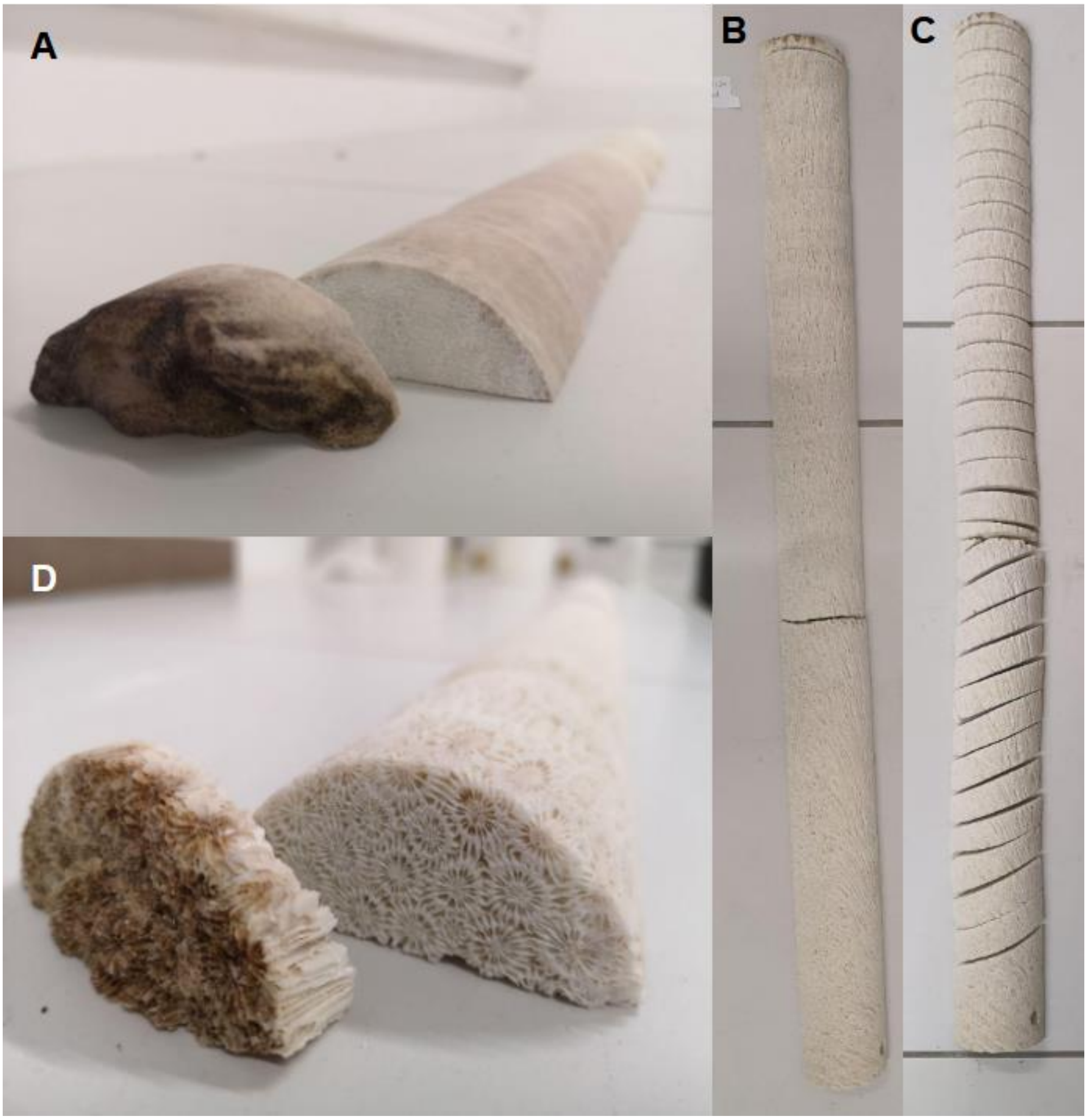

Figure. S1. Photographs of the coral skeleton cores. **A.** Halved coral core of *Porites lobata* including the tissue fragment. **B.** Halved coral core of *Diploastrea heliopora* prior to sectioning into 5-year fragments. **C.** *Diploastrea heliopora* coral core after sectioning into 5-year fragments, note the change in growth axis within the core. **D.** Halved coral core of *Diploastrea heliopora* including the tissue fragment. Picture credit: Jessica Reichert.

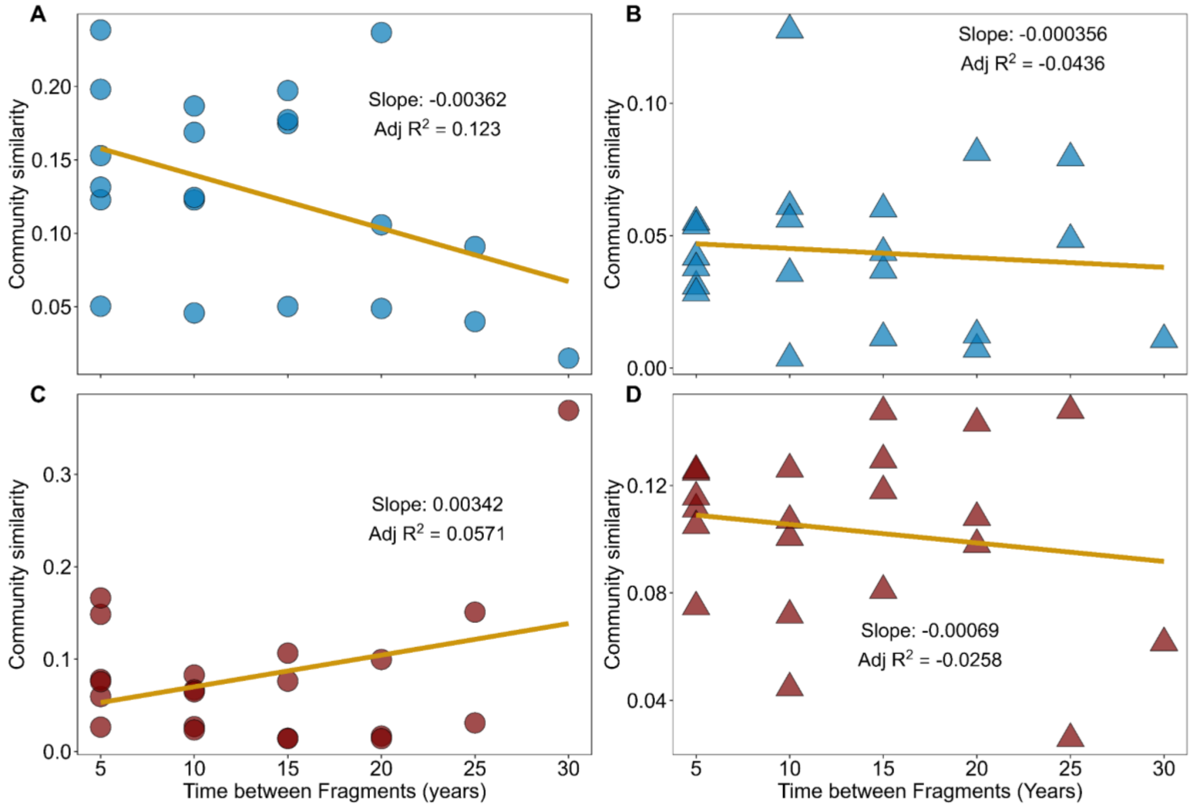

Figure S2. Distance-decay linear regression fitted on community similarity (Bray-Curtis distance matrix) and coral fragment age (Euclidean distance matrix). *Porites lobata* core from **A.** Palau and **B.** Papua New Guinea. *Diploastrea heliopora* core from **C.** Palau and **D.** Papua New Guinea.

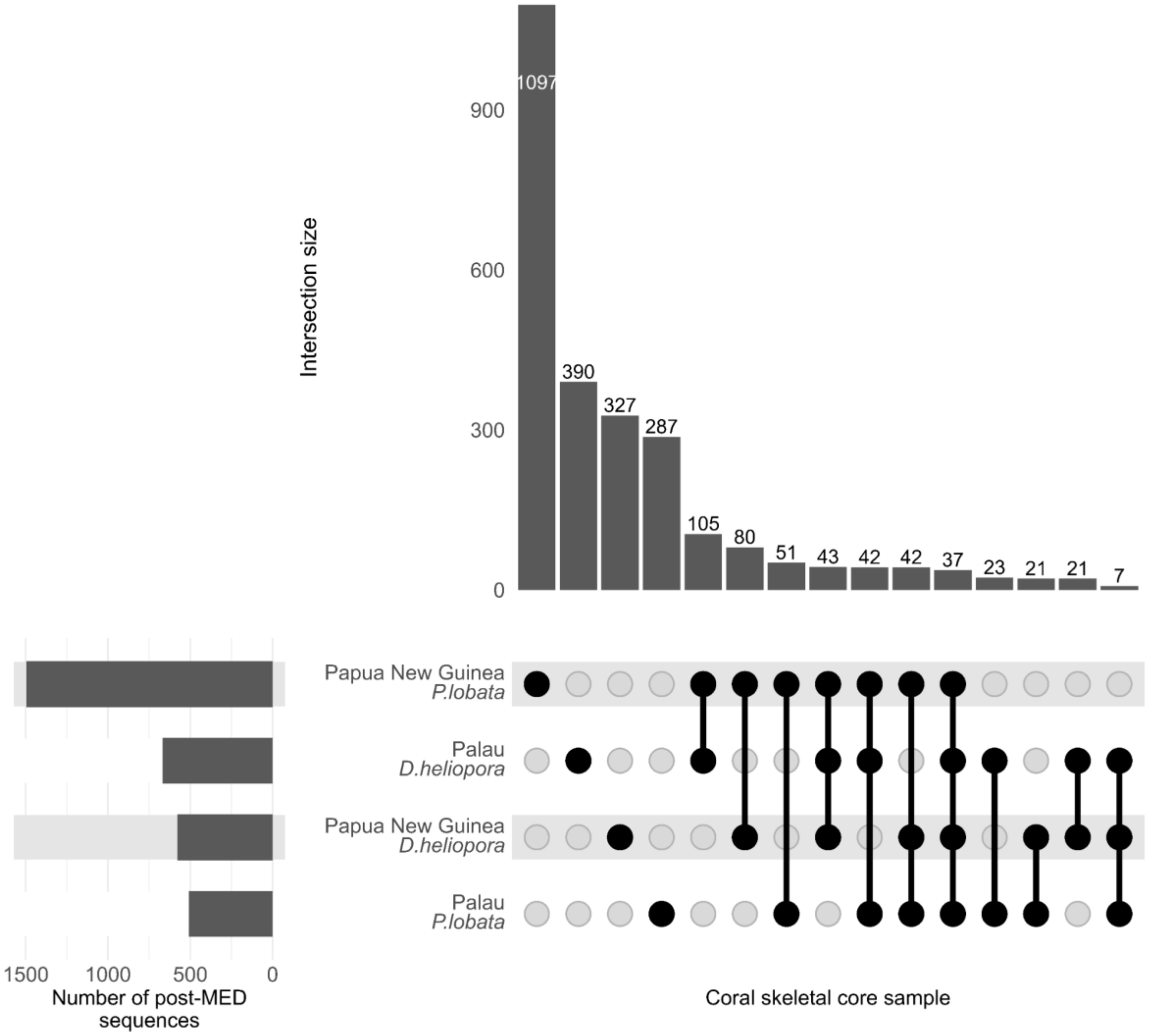

Figure S3. Number of shared post-MED sequences between *P. lobata* and *D. heliopora* skeletal cores sampled in Palau and Papua New Guinea. The intersection size (vertical bar plot) represents the number of shared sequences of a set of skeletal core samples represented by filled points.

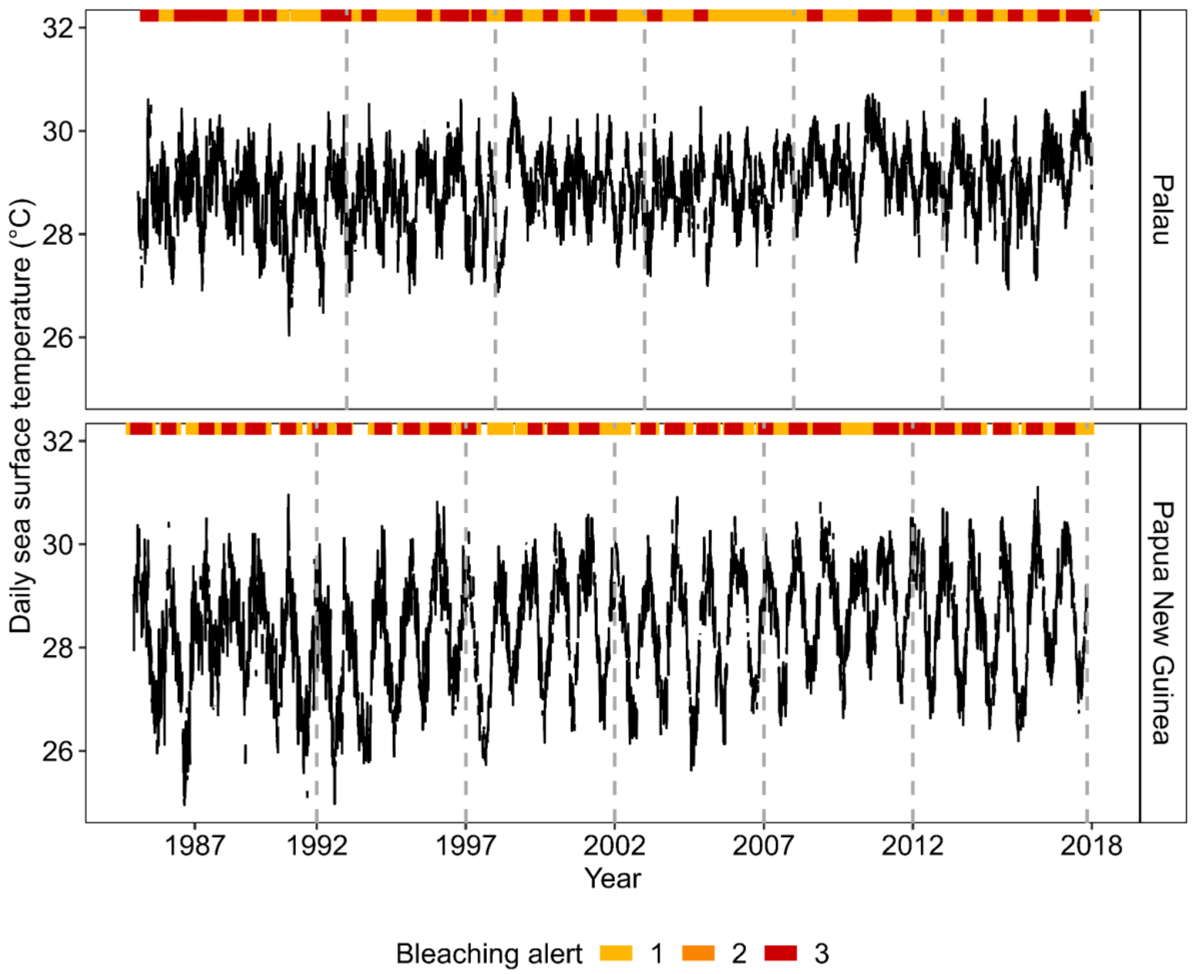

Figure S4. Daily sea surface temperature and days with bleaching alerts for the sampling locations obtained from the NOAA Coral Reef Watch database. The records range from 1985 to 2018.
